# Recommendations to conduct and report systematic reviews in medical literature: a scoping review

**DOI:** 10.1186/s12874-019-0870-1

**Published:** 2019-12-11

**Authors:** Ana Penedones, Carlos Alves, Francisco Batel-Marques

**Affiliations:** 1 Pharmacovigilance Unit of Coimbra (UFC), Centre for Health Technology Assessment and Drug Research (CHAD), AIBILI, Coimbra, Portugal; 2 Laboratory of Social Pharmacy and Public Health, School of Pharmacy, University of Coimbra, Coimbra, Portugal

**Keywords:** Review literature as topic, Epidemiology, Evidence-based medicine

## Abstract

**Background:**

This scoping review aims to identify, review and characterize the published recommendations to conduct and/or to report a systematic review in medical interventions area.

**Methods:**

A search was carried out in PubMed, EMBASE and Cochrane Library databases, using systematic reviews search filters. The search comprises all recommendations to conduct and/or report a systematic review. Data on methods were extracted from each recommendation. A descriptive analysis was performed.

**Results:**

Eighty-three recommendations were identified. Approximately 60% of retrieved references were published in the last 6 years. Recommendations to both conduct and report a systematic review were issued in 47% studies. The guidance presented in each recommendation to conduct and/ or report a systematic review varied. Almost 96% of the recommendations offer guidance on systematic review methods section. The need and time for updating was only recommended in 29% of recommendations. Forty percent of recommendations endorsed their methods to any subject related to medical interventions. Half of the studies did not specify the design of studies to be included in a systematic review.

**Conclusions:**

Several recommendations to conduct and/or report a systematic review were published and offered different guidance. Further research on the impact of such heterogeneity can improve systematic reviews quality.

## Background

A systematic review aims to collect evidence from the research literature, using systematic and explicit methods, to answer a clearly formulated research question [1, 2]. It is a rigorous methodology to identify, select, assess methodological quality, analyze and discuss relevant studies [1, 2]. These characteristics distinguish a systematic review from other type of reviews, since the systematic appraisal of studies based on methodological quality can provide useful information to the clinical decision process, regulatory decisions, and clinical guidelines [1–3].

The first clinical systematic review was published in 1955 in the Journal of the American Medical Association (JAMA) [4]. At the end of 80s, the first systematic review and meta-analysis in the health field, entitled ‘Effective Care during Pregnancy and Childbirth’, was published [5]. In the year of 2015, approximately 950 reviews were published only on the Cochrane Database of Systematic Reviews [6].

Several groups have been dedicated to develop and improve systematic reviews methodology. In the 90s, the Cochrane Collaboration was created with the goal of “prepare, maintain, and disseminate systematic reviews” [7]. Later, this group published the “Cochrane Handbook for Systematic Reviews of Interventions” [8]. Other main groups developed their own guidance on systematic reviews, such as the Centre for Reviews and Dissemination (CRD) which published “Systematic Reviews CRD’s guidance for undertaking reviews in healthcare” [9]; and The Joanna Briggs Institute (JBI) which developed the “The JBI Reviewer’s Manual” [10]. In 1999, a group developed guidance on the reporting of meta-analysis (the QUOROM, QUality Of Reporting Of Meta-analyses) [11]. Ten years later, this guidance was updated and included recommendations on the reporting of systematic reviews (PRISMA, Preferred Reporting Items of Systematic reviews and Meta-Analyses) [11]. Since then, the PRISMA group has been developing specific recommendations on the reporting of systematic reviews, such as abstracts, equity, harms, diagnostic test accuracy, among others [11]. The PRISMA Statement has been endorsed by several scientific journals as the recommend guidance to report a systematic review [11].

The selection of a methodology will depend on the research question and type of review [8–10]. It is recognized that the majority of the recommendations to conduct a systematic review follow four primary steps: 1) review of the literature; 2) selection of criteria to include studies for analysis; 3) extraction of the data from the selected studies; and 4) analysis of the extracted data [8–10]. Since several recommendations are available to help to conduct and/or reporting a systematic review, the knowledge of each recommendation along with the specificity and individuality of each area (for instance by disease or by type of intervention) could define the best methodology to adopt on the conduct and/or report of a systematic review.

The objective of this scoping review is to identify, review and characterize the recommendations available in healthcare literature to conduct and/or to report a systematic review.

## Methods

This scoping review was developed according to the recommendations of The Joanna Briggs Institute Reviewer’s Manual – Methodology for Scoping Reviews [10].

### Literature search and data selection

A search was carried out in PubMed (https://www.ncbi.nlm.nih.gov/pubmed/), EMBASE (https://www.embase.com/), and Cochrane Library (http://cochranelibrary-wiley.com/cochranelibrary/search/advanced) databases. The databases were searched since its inception until July 17, 2018. The search terms comprised systematic reviews methodology. A filter was applied to restrict the search to English articles. The reference list of all identified articles was also hand searched for additional studies. The literature search and search strategy are listed in the Appendix 1.

Articles were selected for inclusion if they meet the following selection criteria: published in English language; conducted in humans; and were recommendations to conduct and/or to report a systematic review of healthcare interventions (e.g., drugs, medical, surgical, behavioral and occupational therapy, and diagnostic testing). Articles describing exclusively the use of qualitative evidence were excluded. Articles such as editorials, letters, commentaries, and abstracts from congresses and articles describing recommendations on how to read or to interpret a systematic review were also excluded. A recommendation could be described in a series of articles or in a single article, this is, in one or more references.

Two researchers independently screened by hand the titles and abstracts and selected full articles for inclusion. Disagreement was resolved by discussion and consensus.

### Data extraction

The following information was extracted independently from each article:

A. Reference, including authors’ names and year of publication
B. Methodological design used to develop a recommendation to conduct and/or report a systematic review, classified between review or consensus study;
C. Name attributed to the recommendation, if applicable;
D. Type of recommendations: to conduct and/or report a systematic review. A recommendation to conduct a systematic review describes the steps to perform it; instead of a recommendation to report a systematic review which describes on how it should be write;
E. Suggested methodology to conduct and/or report a systematic review; the methodological recommendations analyzed were divided into the following sections: 1) introduction; 2) identification of the research question; 3) definition of research protocol; 4) definition of eligibility criteria; 5) execution of literature search; 6) identification of sources of information; 7) data selection; 8) data extraction; 9) risk of bias/methodological quality assessment; 10) data analysis; 11) presentation of results; 12) interpretation of results; 13) discussion/conclusion of results; 14) need and time for updating; 15) helpful material;
F. The subject of the methods issued, for example by disease or study area;
G. Type of studies to be included in the systematic review, for instance, randomized controlled trials (RCT), observational studies, among others;
H. Study group’s name, this is who issued the methodology, if applicable.

### Data analysis

Data were analyzed using descriptive statistics. Statistical analyses were conducted with Microsoft Excel 2010 (Microsoft Corporation, Santa Rosa, CA).

## Results

**Fig. 1:**
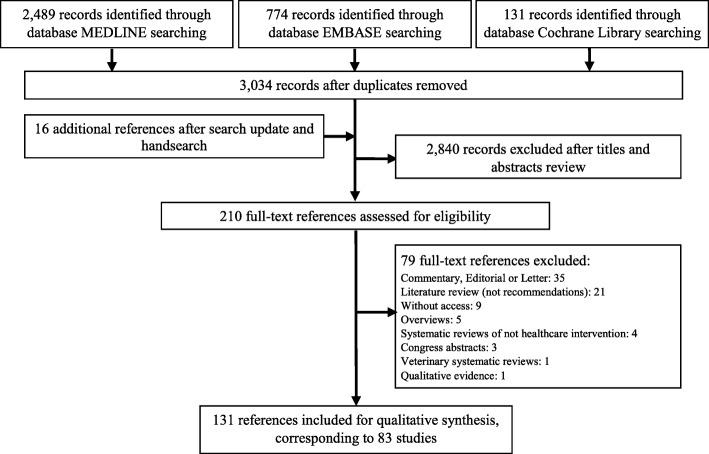
Flow diagram of Literature search

A total of 3034 potentially relevant references were yielded from literature search. Twenty additional references were identified. Based on the inclusion criteria, 210 references were selected for full-text further inclusion. A final sample of 131 references covering 83 different recommendations met the inclusion criteria (some recommendations were described in a series of references, this is, in more than one article). The selection of references is shown in Fig. 1. The references of the included and excluded studies are listed in the Additional file 1.

Sixty-three percent (*n* = 83/131) of retrieved articles were published since 2012 (Appendix 2).

Sixty out of 83 (72%) recommendations were developed through a review study, whereas 23 (28%) as a consensus study.

**Fig. 2:**
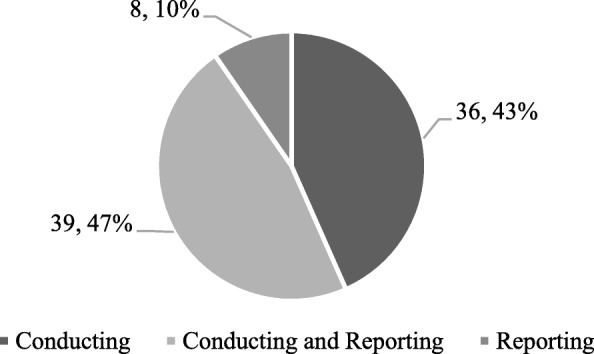
Type of recommendations, to conduct and/or report a systematic review

Guidance to conduct and report a systematic review were issued in 39 (47%) recommendations. Only 10% (*n* = 8/83) of recommendations described guidance on how to report a systematic review. The type of recommendations is described in Fig. 2. A detailed description of the recommendations is presented in Appendix 2.

Table 3 describes the methods’ sections to conduct and/or report a systematic review for which the recommendations offered guidance.

**Table 3:** Methods to conduct and/or report a systematic review as suggested in each study

Methods to conduct and/or report a systematic review	N (%)^¥^
Introduction (background, purpose)	38 (46%)
Identification of the research question	78 (94%)
Definition of research protocol	59 (71%)
Definition of eligibility criteria	81 (98%)
Execution of literature search	79 (95%)
Identification of sources of information	76 (92%)
Data selection	79 (95%)
Data extraction	73 (88%)
Risk of bias/methodological quality assessment	81 (98%)
Data analysis	82 (99%)
Presentation of results	75 (90%)
Interpretation of results	66 (80%)
Discussion/conclusion of results	60 (72%)
Need and time for updating	24 (29%)
Helpful material (tables, graphics, methodological quality assessment scales, flowcharts)	30 (36%)

^**¥**^The table presents the number of recommendations that issued each specified method

Methodological steps to analyze data, assess risk of bias/ methodological quality and define eligibility criteria were addressed by most of the recommendations (between 98 and 99% of recommendations). The definition of an a priori research protocol was less often recommended (59 (71%) recommendations). An orientation about how to prepare an introduction, including the background and purpose of systematic review, was only comprised in 38 (46%) recommendations. Guidance about the need and time for updating the systematic review were included in only 24 (29%) recommendations. Thirty (36%) recommendations made available supportive material to conduct and/or report a systematic review, such as tables, graphics, methodological quality assessment scales, and/or flowcharts.

Thirty-three (39.8%) recommendations endorsed their methods to any subject related to healthcare interventions. Twenty (24%) recommendations are specific of a clinical subject, such as cardiology, pain, nephrology, and sports medicine and orthopedic surgery. Eleven (13.2%) recommendations addressed guidance on the conduction and/or report of systematic reviews about investigation procedures. Other subjects, such as economic evaluation, were also studied. The subject of recommendations is presented in Table 4.

**Table 4:** Subject of the methods issued to conduct and/or report a systematic review by each study

Subject of methods	N (%)
Any	33 (39.8%)
Any, Adverse effects, Economic evidence, Qualitative research, Public health, and health promotion^**¥**^	1 (1.2%)
Any, Diagnostic tests, Prognostic tests, Public health interventions, Adverse Effects, Economic evaluations, Qualitative evidence^**¥**^	1 (1.2%)
Any, Qualitative evidence, Quantitative evidence, Economic evidence, Textual and non-research evidence, Text and opinion data^**¥**^	1 (1.2%)
Clinical Subject	20 (24.0%)
Cardiology	4 (4.8%)
Pain	2 (2.4%)
Nephrology	2 (2.4%)
Sports medicine and orthopedic surgery	2 (2.4%)
Geriatric	1 (1.2%)
Neck and back pain, and related spinal disorders	1 (1.2%)
Nutrition	1 (1.2%)
Ophthalmology	1 (1.2%)
Pathology	1 (1.2%)
Plastic and Reconstructive Surgery	1 (1.2%)
Pregnancy and childcare	1 (1.2%)
Radiology	1 (1.2%)
Tuberculosis	1 (1.2%)
Urology	1 (1.2%)
Investigation procedures subject	11 (13.2%)
Diagnostic test	5 (6.0%)
Diagnostic test and prognostic test	2 (2.4%)
Medical tests, genetic tests, and prognostic tests	1 (1.2%)
Radiography	1 (1.2%)
Surgical procedures	1 (1.2%)
Toxicology	1 (1.2%)
Other healthcare interventions	8 (9.6%)
Rehabilitation	3 (3.6%)
Nursing practice	2 (2.4%)
Pediatric practice nursing	1 (1.2%)
Physiotherapy	1 (1.2%)
Occupational therapy	1 (1.2%)
Others	8 (9.6%)
Economic	2 (2.4%)
Harms	2 (2.4%)
Anatomy	1 (1.2%)
Complex multi-component health care interventions	1 (1.2%)
Patient-reported outcome measures	1 (1.2%)
Prediction model performance	1 (1.2%)
**Total**	**83 (100%)**

^**¥**^Each recommendation develop methods for several types of systematic reviews

Fifty-two (62.7%) recommendations did not specify the studies’ design to be included in the systematic review. Among those addressing this issue, clinical trials and randomized controlled trials were the type of study preferred to conduct a systematic review (*n* = 9; 10.8%). The type of studies eligible for inclusion in the systematic review is presented in Table 5.

**Table 5:** Type of studies eligible for inclusion in the systematic review recommended by each study

Type of studies	N (%)
Any	52 (62.7%)
RCT	6 (7.2%)
Diagnostic test studies	6 (7.2%)
Clinical trials	3 (3.6%)
Economic evaluations	2 (2.4%)
Diagnostic and prognostic studies	2 (2.4%)
Adverse events	1 (1.2%)
Anatomical studies	1 (1.2%)
Etiology studies	1 (1.2%)
Evidence on equity	1 (1.2%)
Medical tests, genetic tests, and prognostic tests	1 (1.2%)
Network meta-analysis	1 (1.2%)
Observational studies	1 (1.2%)
Observational studies reporting prevalence and cumulative incidence data	1 (1.2%)
Protocols	1 (1.2%)
RCT, observational studies, diagnostic tests^a^	1 (1.2%)
Studies of older people	1 (1.2%)
Validation studies	1 (1.2%)
**Total**	**83 (100%)**

^a^The recommendation developed methods to conduct a systematic review with each type of study

Sixty-seven percent of the recommendations were issued by individual groups/authors, without being affiliated with any particular organization researching in methods used in systematic review. Some organizations such as The Joanna Briggs Institute, The Cochrane Collaboration, The Centre for Reviews and Dissemination, and the Agency for Healthcare Research and Quality also developed, at least one, recommendation to conduct and/or report a systematic review. The distribution of the recommendations by study groups is presented in Table 6.

**Table 6:** Study groups, who issued the methods to conduct and/or report a systematic review

Study group	N (%)
PRISMA	7 (8.4%)
The Joanna Briggs Institute	6 (7.2%)
The Cochrane Collaboration	4 (4.8%)
AHRQ	3 (3.6%)
AHRQ and JGIM	1 (1.2%)
American Heart Association	1 (1.2%)
Centre for Reviews and Dissemination, University of York	1 (1.2%)
COSMIN	1 (1.2%)
European Association of Urology	1 (1.2%)
World Association of Laser Therapy	1 (1.2%)
Other (individual groups/authors)	57 (67.5%)
Total	83 (100%)

PRISMA: Preferred Reporting Items for Systematic Reviews and Meta-Analysis; AHRQ: Agency for Healthcare Research and Quality; JGIM: Journal of General Internal Medicine; COSMIN: Consensus-based Standards for the selection of health Measurement Instruments.

## Discussion

In the last years, several organizations and individual groups have published recommendations to conduct and/or report a systematic review. In general, they can be applied to study any healthcare intervention, combining different types of evidence. The recommendations focus on the methods of the systematic review.

In the present study, only orientations about systematic review were identified, characterized and reviewed; however, some of the recommendations include guidance to conduct and/or to report a meta-analysis.

More than half of the recommendations evaluated in this study were published in the last 6 years. The volume of information and new studies is growing. In MEDLINE, one of the largest databases of medical literature, more than 8 million articles were indexed in 23 years [12]. In addition, systematic reviews synthesize several types of study, such as network meta-analysis, adverse events, economic studies, among others [8–10]. The need for specific recommendations addressing this type of studies was also increased over time. This can explain the growth of certain organizations such as the Cochrane Collaboration or the JBI and the development of such specific recommendations [8–10]. Moreover, regulatory authorities required a compilation of various individual studies in the health technology assessment, for instance in market access [13], in its re-evaluation and to monitoring its benefit-risk ratio [14]. A systematic review becomes a recognized need to support informed decisions in medicine. A study by Bastian et al. estimated that 11 systematic reviews are published per day [15]. Therefore, guidance on how to conduct and/or report a systematic review of any kind becomes essential [16].

According to The Grading of Recommendations Assessment, Development and Evaluation (GRADE) working group, the development of a recommendation should include a review of all existing evidence on the research question and an evaluation of this data by a panel of experts. After that, a consensus is achieved on which steps a recommendation should follow [17]. However, among the recommendations analyzed in this study, approximately 70% were published under a review study, and only 30% were developed based on a consensus of a panel of experts.

Some organizations were created to study and develop guidance on the best synthesis of different types of information and, thereafter, the elaboration of systematic reviews [8–10]. In this review, nearly 32% of the recommendations were issued by these organizations. The other 68% were issued by individual groups/authors. The growth in publication of scientific studies reflects the need to conduct methodological well/structured reviews of the literature [12]. This may also result in the increase of recommendations to conduct and/or report systematic reviews, particularly recommendations for a specific area, such as safety or economic evaluations.

A recommendation to conduct and/or report a systematic review should list and detail all fundamental steps to help authors to write, or scientific journals to appraise a systematic review. From the recommendations characterized in this review, between 88 and 99% developed guidance on methods (from the definition of eligibility criteria to data analysis). The elaboration and publication of a systematic review protocol improves transparency and avoids duplication of work [8]. In this review, only half of the recommendations addressed the elaboration of a protocol. Nowadays, there are several ways to publish a systematic review protocol, such as registration in PROSPERO (International prospective register of systematic reviews) or publication of the protocol in peer-reviewed journals [3]. However, a significant proportion (44%) of the recommendations issuing the elaboration of a protocol was published during the last 4 years (since 2015). This becomes interesting since it shows the importance of continuing to address this step in the recommendations to conduct and/or report a systematic review. Despite the majority of the recommendations offer orientations on how to define the research question, the elaboration of the Introduction was the step less described. Describing the background and the purpose of the systematic review may help readers to understand the research question and make a most properly judgement of the results, increasing the systematic review quality [3, 12]. Approximately 76% of the recommendations present guidance on the interpretation and discussion of the results. This proportion seems to be low since systematic reviews’ main goals are to inform and help the interested parties in the decision-making processes. ‘Need and time for updating’ was the step less recommended. Systematic reviews are constantly out of date with new evidence published every day [18]. Recently, the panel for updating guidance for systematic reviews (PUGs) group had illustrated the importance of update systematic reviews and developed some guidance that can help authors and readers to understand when to update a systematic review [19]. Moreover, in 2014, the concept of living systematic review emerged [20]. This intended to continually updated a systematic review (of any type). It predisposes a periodic search and the constant update since new data arises [20]. Nonetheless, despite some guidance for updating systematic reviews are available, it is still necessary to include this step in the recommendations to conduct and/ or report a systematic review.

In this review, all recommendations presented some differences in methodology which may lead to some bias, such as reporting bias. These bias reflects the influence on their reporting, which can lead to a misunderstanding of the results [21]. If the recommended methodology to conduct a systematic review is not clear enough, the results and conclusions of the produced systematic review could be flawed, limiting its importance and objective [12]. Such methodological impairments may compromise the comparability of systematic reviews addressing the same research question, eligibility criteria, search criteria, and time of research, but which follow different recommendations [22]. Therefore, their results, based on the same studies, may be biased and presented in different ways. Thus, each systematic review could present its own conclusions, introducing confounding in health decision-making [22].

Eight recommendations were specifically developed to address the reporting of systematic reviews. Seven of these recommendations were developed by PRISMA working group. Currently, PRISMA has becoming a wide-scale adopted guideline, used by authors to report and by scientific journals to appraise a systematic review [11]. The PRISMA, a guideline created to increase the quality of reporting a systematic review, aims at enhancing transparency, reliability, and ease of reading [11]. Several studies demonstrated the poor quality of systematic reviews when they are not compliant with a reporting guideline [11]. Despite the publication and dissemination of PRISMA, there are several studies showing the suboptimal compliance to this guidance when reporting a systematic review [23, 24]. Moreover, one of the PRISMA extensions, PRISMA of Diagnostic Test Accuracy (PRISMA-DTA, has recently being update in order to improve the reporting in systematic reviews of this type [25].

The safety of healthcare interventions is of major importance. The knowledge of their safety profile should be continuously updated to keep healthcare professionals, consumers, and healthcare regulators informed [8–10]. To characterize the safety profile, several types of information provided by distinct sources need to be consulted. In opposite to efficacy data, safety data is mainly obtained from post-marketing surveillance data sources, which comprises several types of studies, such as post-marketing clinical trials, observational studies, case reports, and spontaneous reports of adverse events [26]. Combining evidence from these several sources presupposes some specific methodology in conducting and/or reporting systematic reviews. This review identified four recommendations addressing how to conduct and/or report systematic reviews of adverse events.

Some other relevant areas related to healthcare interventions were also taken into consideration, such as economic evaluations of healthcare interventions. Expenditure with pharmaceuticals may account for a significant amount of health spending, depending on the countries [27]. Thereafter, pharmacoeconomic studies become essential in supporting the appraisal of medical interventions, medicines, and their market access [13]. A systematic review of these studies is important for healthcare policymaking [28]. However, the role of systematic review to synthesize economic evaluations has been questioned [29]. Not only due to the specific design of economic evaluations, such as type of analysis, perspective adopted, among others, but also because economic evaluations already synthesize information [29]. Thus, the elaboration of specific recommendations to conduct and/or report a systematic review of economic evaluations may be valuable.

Almost half of the analyzed recommendations did not specify the design of studies to be included in a systematic review. Some recommendations only endorse the inclusion of randomized controlled trials, because of its classification such as the highest level of evidence [30]. Nonetheless, the type of studies selected must reflect the objective of the systematic review. In a systematic review evaluating the effectiveness of investigation procedures, such as diagnostic tests, studies evaluating the accuracy of diagnostic tests must be chosen [31]. Naturally, the methodological quality level of the evidence chosen will be varied. However, an evaluation of the risk of bias or methodological quality of the studies included must be conducted. Hereafter, the results of this evaluation must be included in the interpretation of the results of the systematic review [3, 8].

This scoping review has several limitations. An a priori protocol was not previously published. The search was conducted according to the PubMed, EMBASE and Cochrane Library databases indexed terms for studies about systematic reviews’ methodology. These indexed terms may not comprise all recommendations published in literature. Despite the combination of these terms with free terms such as “methods”, the search strategy may not be comprehensive and some references may not have been included. References from other languages than English were not analyzed. In addition, grey literature was not searched. This could lead to the exclusion of some recommendations. Therefore, the results must be interpreted carefully. This review offers an overview of what is published and does not intend to address criticism or influence the choice of a specific recommendation. The preliminary results of this study were presented at ISPOR Europe 2018: New Perspectives for Improving twenty-first Century Health Systems [32].

## Conclusions

Several recommendations to conduct and/or report a systematic review are available to combine evidence from diverse healthcare areas. Such recommendations differ in some methodological aspects. Further research on the implications of such heterogeneity seems important, in order to guarantee systematic review transparency, quality and its role in healthcare.

### Supplementary information

**Additional file 1:** List of references from included and excluded studies.

## Data Availability

All data generated or analyzed during this study are included in this published article [and its supplementary information files].

## Appendix 1

**Table 1:** Search literature and Search strategy

Search	Equation	Results
*PUBMED*		
#1	“Review Literature as Topic”[Majr]	4189
#2	“methods”[MeSH Terms] OR “methods”[All Fields] OR “method”[All Fields]	7,857,103
#3	#1 AND #2	2639
#4	#3 AND English [lang]	2489
*COCHRANE LIBRARY*		
#1	“Review Literature as Topic”[Majr]	180
#2	“methods”[MeSH Terms] OR “methods”[All Fields] OR “method”[All Fields]	638,931
#3	#1 AND #2	131
#4	#3 AND English [lang]	131
*EMBASE*		
#1	‘systematic review (topic)’/mj	800
#2	‘systematic review (topic)’/mj AND [english]/lim	774

Date: Since databases’ inception until July 17, 2018

Definitions on search terms according to databases websites:

“Review Literature as Topic” - Works about published materials which provide an examination of recent or current literature. These articles can cover a wide range of subject matter at various levels of completeness and comprehensiveness based on analyses of literature that may include research findings. The review may reflect the state of the art and may also include reviews as a literary form

“Methods” - A series of steps taken in order to conduct research

‘systematic review (topic)’ - used for items that discuss systematic reviews

Databases of websites:

PubMed: *https://www.ncbi.nlm.nih.gov/pubmed/*

Cochrane Library: *http://cochranelibrary-wiley.com/cochranelibrary/search/advanced*

EMBASE: *https://www.embase.com*

## Appendix 2

**Table 2:** Detailed description of the recommendations to conduct and/or report a systematic review

Recommendation (citations)	Type of study	Purpose	Subject	Organization	Studies included
Aromataris E and Pearson A, 2014 [1] Stern C et al., 2014 [2] Aromataris E and Riitano D, 2014 [3] Porritt K et al., 2014 [4] Munn Z et al., 2014 [5] Robertson-Malt S, 2014 [6]	Review	Conducting and Reporting	Nursing practice	The Joanna Briggs Institute	Any
Brown PA et al., 2012 [7]	Review	Conducting	Rehabilitation research	–	Any
Campbell JM et al., 2015 [8]	Consensus study	Conducting	Diagnostic test	The Joanna Briggs Institute	Diagnostic test accuracy studies
Centre for Reviews and Dissemination’s guidance, 2009 [9]	Review	Conducting and reporting	Any, Diagnostic tests, Prognostic tests, Public health interventions, Adverse Effects, Economic evaluations, Qualitative evidence	Centre for Reviews and Dissemination, University of York	Any
Chalmers I et al., 1993 [10]	Review	Conducting	Pregnancy and childcare	–	RCT
Chou R et al., 2018 [11]	Consensus study	Conducting and Reporting	Harms	AHRQ	Adverse events
Cochrane Back and Neck Group, 2003, 2009, 2014, 2015 [12–15]	Consensus study	Conducting and reporting	Neck and back pain, and related spinal disorders	The Cochrane Collaboration	Clinical trials
Cochrane Diagnostic Test Accuracy Working Group, 2008 [16]	Consensus study	Conducting and reporting	Diagnostic accuracy	The Cochrane Collaboration	Diagnostic test accuracy studies
Cochrane Handbook for systematic reviews of interventions, 2011 [17]	Review	Conducting and reporting	Any, Adverse effects, Economic evidence, Qualitative research, Public health and health promotion	The Cochrane Collaboration	Any
Cook DA and West CP, 2012 [18]	Review	Conducting and Reporting	Any	–	Any
COSMIN guideline, 2018 [19]	Consensus study	Conducting	Patient-reported outcome measures	COSMIN	Any
Cronin P et al., 2018 [20]	Review	Conducting	Diagnostic accuracy (imaging)	–	Diagnostic imaging studies
Cronin P, 2008 [21]	Consensus study	Conducting and Reporting	Any	–	Any
Crowther DM, 2013 [22]	Review	Conducting and Reporting	Any	–	Any
Crowther M et al., 2010 [23]	Review	Conducting	Any	–	Any
Da Costa BR and Jüni P, 2014 [24]	Review	Conducting and Reporting	Any	–	RCT
Davis D, 2016 [25]	Review	Conducting and Reporting	Any	–	Any
de Vet HC et al., 2005 [26]	Consensus study	Conducting	Physiotherapy	–	RCT
Debray TPA et al., 2017 [27]	Review	Conducting and Reporting	Prediction model performance	–	Validation studies
Dijkers MP et al., 2012 [28]	Review	Conducting and reporting	Rehabilitation research	–	Any
Fares M et al., 2016 [29]	Review	Conducting	Cardiology	–	Any
Gomersall et al., 2015 [30]	Consensus study	Conducting	Economic	The Joanna Briggs Institute	Economic evaluations
Goodacre S, 2009 [31]	Review	Conducting	Any	–	Any
Guise JM et al., 2014 (a) [32] Guise JM et al., 2017 (b) [33] Kelly MP et al., 2017 [34] Butler M et al., 2017 [35] Viswanathan M et al., 2017 [36] Pigott T et al., 2017 [37] PRISMA-CI: Guise JM et al., 2017 (c) [38]; Guise JM et al., 2017 (d) [39]	Consensus study	Conducting and Reporting	Complex multicomponent health care interventions	AHRQ	Any
Haase SC, 2011 [40]	Review	Conducting and Reporting	Any	–	Any
Harris JD et al., 2014 [41]	Review	Conducting and Reporting	Sports medicine and orthopedic surgery	–	Any
Henderson LK et al., 2010 [42]	Consensus study	Conducting and Reporting	Nephrology	The Cochrane Collaboration	Any
Hoffmann S et al., 2017 [43]	Review	Conducting and Reporting	Toxicology	–	Any
Hopp L and Rittenmeyer L, 2015 [44]	Review	Conducting and Reporting	Any	–	Any
Joanna Briggs Institute Reviewers’ Manual, 2017 [45]	Consensus study	Conducting and reporting	Any, Qualitative evidence, Quantitative evidence, Economic evidence, Textual e non-research evidence, Text and opinion data	JBI	Any
Jones T and Evans D, 2000 [46]	Review	Conducting	Nursing practice	–	RCT
Kalra R et al., 2017 [47]	Review	Conducting and Reporting	Cardiology	–	Any
Kelley BP and Chung KC, 2018 [48]	Review	Conducting and Reporting	Plastic and Reconstructive Surgery	–	Any
Khan KS et al., 2003 [49]	Review	Conducting	Any	–	Any
Khan KS, 2005 [50]	Review	Conducting	Diagnostic test accuracy	–	Diagnostic test accuracy studies
Koretz RL and Lipman TO, 2017 [51]	Review	Conducting and Reporting	Any	–	RCT
Kranke P, 2010 [52]	Review	Conducting	Any	–	Clinical trials
Leeflang MM, 2014 [53]	Review	Conducting	Diagnostic test accuracy	–	Diagnostic test accuracy
Lipp A, 2003 [54]	Review	Conducting and Reporting	Surgical face marks*	–	RCT
Liu Z et al., 2013 [55]	Review	Conducting	Diagnostic test and prognostic test	–	Diagnostic and prognostic test accuracy evaluations
Manchikanti L et al., 2009 (a) [56] Manchikanti L et al., 2009 (b) [57] Manchikanti L et al., 2009 (c) [58]	Review	Conducting and Reporting	Interventional pain management	–	RCT, Observational studies, Diagnostic tests
Marchevsky AM and Wick MR, 2015 [59]	Review	Conducting	Pathology	–	Any
Marshall G and Sykes AE, 2011 [60]	Review	Conducting and Reporting	Radiography	–	Any
Matchar DB, 2012 [61] Samson D and Schoelles KM, 2012 [62] Segal, 2012 [63] Relevo R, 2012 [64] Santaguida PL et al., 2012 [65] Hartmann KE et al., 2012 [66] Singh S et al., 2012 [67] Trikalinos TA et al.,2012 (a) [68] Trikalinos TA and Balion CM, 2012 [69] Trikalinos TA et al., 2012 (b) [70] Jonas DE et al., 2012 [71] Rector TS et al., 2012 [72]	Review	Conducting	Medical tests, Genetic tests, Prognostic tests	AHRQ and JGIM	Medical tests, genetic tests, and prognostic tests
Menzies D, 2011 [73]	Review	Conducting and Reporting	Tuberculosis	–	Any
Methodology of the European Association of Urology, 2018 [74]	Consensus study	Conducting	Urology	European Association of Urology	Any
Methods Guide for Effectiveness and Comparative Effectiveness Reviews, 2014 [75]	Consensus study	Conducting and reporting	Any	AHRQ	Any
Milner KA, 2015 [76]	Review	Conducting and Reporting	Any	–	Any
Moola S et al., 2015 [77]	Consensus study	Conducting	Any	The Joanna Briggs Institute	Etiology studies
Munn Z et al., 2015 [78]	Consensus study	Conducting	Any	The Joanna Briggs Institute	Observational studies reporting prevalence and cumulative incidence data
Neely JG et al., 2010 [79]	Review	Conducting and Reporting	Any	–	Any
Nguyen NH and Singh S, 2018 [80]	Review	Conducting and Reporting	Any	–	Any
Nicholson PJ, [81]	Review	Conducting and Reporting	Occupational therapy	–	Any
Noordzij M et al., 2011 [82]	Review	Conducting	Nephrology	–	Any
Pollock A and Berge E, 2018 [83]	Review	Conducting	Stroke (rehabilitation)	–	Any
PRISMA, 2009, 2010 [84–93]	Consensus study	Reporting	Any	PRISMA	Any
PRISMA Harms, 2016 [94]	Consensus study	Reporting	Adverse events	PRISMA	Any
PRISMA-DTA, 2018 [95]	Consensus study	Reporting	Any	PRISMA	Diagnostic Test Accuracy Studies
PRISMA-E, 2012, 2015, 2016 [96–98]	Consensus study	Reporting	Any	PRISMA	Evidence on equity
PRISMA-IPD, 2015 [99]	Consensus study	Reporting	Any	PRISMA	Any
PRISMA-NMA, 2015, 2016 [100,101]	Consensus study	Reporting	Any	PRISMA	Network meta-analysis
PRISMA-P, 2015 [102,103]	Consensus study	Reporting	Any	PRISMA	Protocols
Ravindran V and Shankar S, 2015 [104]	Review	Conducting and Reporting	Any	–	Any
Rew L, 2011 [105]	Review	Conducting	Pediatric nursing	–	Any
Riesenberg LA and Justice EM, 2014 [106, 107]	Review	Conducting	Any	–	Any
Rudnicka AR and Owen CG, 2012 [108]	Review	Conducting	Ophthalmology	–	Any
Sambunjak D and Franić M, 2012 [109]	Review	Conducting	Orthopedic surgery	–	Any
Sayers A, 2008 (a) [110] Sayers A, 2007 (b) [111] Sayers A, 2008 (c) [112] Sayers A, 2007 (d) [113]	Review	Conducting	Any	–	Any
Schweizer ML and Nair R, 2017 [114]	Review	Conducting and reporting	Any	–	Any
Scientific Statement from the American Heart Association, 2017 [115]	Consensus study	Conducting	Cardiac Prevention and Treatment	American Heart Association	Any
Shenkin SD et al., 2017 [116]	Review	Conducting and Reporting	Healthcare of older people	–	Studies of older people
Sousa MR and Ribeiro AL, 2009 [117]	Review	Conducting and reporting	Diagnostic and prognostic	–	Diagnostic and Prognostic Studies
Standards of World Association of Laser Therapy, 2006 [118]	Consensus study	Conducting and Reporting	Low-Level Laser Therapy for Musculoskeletal Pain and Disorders	World Association of Laser Therapy	Clinical trials
Staunton M, 2007 [119] Halligan S and Altman DG, 2007 [120]	Review	Conducting	Radiology	–	Any
The EBA process, 2016 [121]	Review	Conducting and Reporting	Anatomy	–	Anatomical studies
Thrift AG, 2010 [122]	Review	Conducting	Any	–	Observational studies
Uman LS, 2011 [123]	Review	Conducting and Reporting	Any	–	Any
Umscheid CA, 2013 [124]	Review	Conducting	Any	–	Any
van Mastrigt GA et al., 2016 [125] Thielen FW et al., 2016 [126] Wijnen B et al., 2016 [127]	Review	Conducting	Economic	–	Economic evaluations
Wanden-Berghe C and Sanz-Valero J, 2012 [128]	Review	Conducting	Nutrition	–	Any
White A and Schmidt K, 2005 [129]	Review	Conducting	Any	–	Any
Wieseler B and McGauran N, 2010 [130]	Review	Reporting	Any	–	Any
Yanagawa B et al., 2018 [131]	Review	Conducting	Cardiac surgery	–	Any

*AHRQ Agency for Healthcare Research and Quality*

## Abbreviations

CRD: Centre for Reviews and Dissemination
GRADE: Grading of Recommendations Assessment, Development and Evaluation
JAMA: Journal of American Medical Association
JBI: The Joanna Briggs Institute
PRISMA: Preferred Reporting Items for Systematic Reviews and Meta-analysis
RCT: Randomized Controlled Trials
