# Supplementary material for: Recommendations to conduct and report systematic reviews in medical literature: a scoping review
Source: BMC Med Res Methodol. 2019 Dec 11;19:234. doi: 10.1186/s12874-019-0870-1 (PMC6907169; doi:10.1186/s12874-019-0870-1)
Supplement: Supplementary file 1 — Additional file 1: List of references from included and excluded studies. [file 12874_2019_870_MOESM1_ESM.docx]

Additional file 1 List of references from included and excluded studies

**Included studies**

1. Aromataris E, Pearson A. The systematic review: an overview. Am J Nurs. 2014;114(3):53-8.
2. Stern C, Jordan Z, McArthur A. Developing the review question and inclusion criteria. Am J Nurs. 2014;114(4):53-6.
3. Aromataris E, Riitano D. Constructing a search strategy and searching for evidence. A guide to the literature search for a systematic review. Am J Nurs. 2014;114(5):49-56.
4. Porritt K, Gomersall J, Lockwood C. JBI's Systematic Reviews: Study selection and critical appraisal. Am J Nurs. 2014;114(6):47-52.
5. Munn Z, Tufanaru C, Aromataris E. JBI's systematic reviews: data extraction and synthesis. Am J Nurs. 2014;114(7):49-54.
6. Robertson-Malt S. Presenting and interpreting findings. Am J Nurs. 2014 Aug;114(8):49-54.
7. Brown PA, Harniss MK, Schomer KG, et al. Conducting systematic evidence reviews: Core concepts and lessons learned. Archives of Physical Medicine and Rehabilitation. 2012;93(8 SUPPL.):S177-S84.
8. Campbell JM, Klugar M, Ding S, et al. Diagnostic test accuracy: methods for systematic review and meta-analysis. Int J Evid Based Healthc. 2015;13(3):154-62.
9. Centre for Reviews and Dissemination, University of York. CRF’s guidance for undertaking reviews in health care. 2009. Available at: https://www.york.ac.uk/media/crd/Systematic_Reviews.pdf (last accessed 08 Oct 2018).
10. Chalmers I, Enkin M, Keirse MJ. Preparing and updating systematic reviews of randomized controlled trials of health care. Milbank Q. 1993;71(3):411-37.
11. Chou R, Baker WL, Bañez LL, et al. Agency for Healthcare Research and Quality Evidence-based Practice Center methods provide guidance on prioritization and selection of harms in systematic reviews. Journal of Clinical Epidemiology. 2018;98:98-104.
12. Furlan AD, Malmivaara A, Chou R, et al. 2015 updated method guideline for systematic reviews in the Cochrane Back and Neck Group. Spine. 2015;40(21):1660-73.
13. Ghogomu EA, Maxwell LJ, Buchbinder R, et al. Updated method guidelines for cochrane musculoskeletal group systematic reviews and metaanalyses. J Rheumatol. 2014;41(2):194-205.
14. Furlan AD, Pennick V, Bombardier C, et al. 2009 updated method guidelines for systematic reviews in the Cochrane Back Review Group. Spine (Phila Pa 1976). 2009;34(18):1929-41.
15. van Tulder M, Furlan A, Bombardier C, et al. Updated method guidelines for systematic reviews in the cochrane collaboration back review group. Spine (Phila Pa 1976). 2003;28(12):1290-9.
16. Leeflang MM, Deeks JJ, Gatsonis C, et al. Systematic reviews of diagnostic test accuracy. Ann Intern Med. 2008;149(12):889-97.
17. Higgins JPT, Green S (editors). Cochrane Handbook for Systematic Reviews of Interventions Version 5.1.0 [updated March 2011]. The Cochrane Collaboration, 2011. Available at: www.handbook.cochrane.org (last accessed 08 Oct 2018).
18. Cook DA, West CP. Conducting systematic reviews in medical education: a stepwise approach. Med Educ. 2012;46(10):943-52.
19. Prinsen CAC, Mokkink LB, Bouter LM, et al. COSMIN guideline for systematic reviews of patient-reported outcome measures. Quality of Life Research. 2018;27(5):1147-57.
20. Cronin P, Kelly AM, Altaee D, et al. How to Perform a Systematic Review and Meta-analysis of Diagnostic Imaging Studies. Academic Radiology. 2018;25(5):573-93.
21. Cronin P, Ryan F, Coughlan M. Undertaking a literature review: a step-by-step approach. Br J Nurs. 2008;17(1):38-43.
22. Crowther DM. A clinician's guide to systematic reviews. Nutr Clin Pract. 2013;28(4):459-62.
23. Crowther M, Lim W, Crowther MA. Systematic review and meta-analysis methodology. Blood. 2010;116(17):3140-6.
24. Da Costa BR, Jüni P. Systematic reviews and meta-analyses of randomized trials: Principles and pitfalls. European Heart Journal. 2014;35(47):3336-45.
25. Davis D. A practical overview of how to conduct a systematic review. Nurs Stand. 2016;31(12):60-71.
26. de Vet HC, Verhagen AP, Logghe I, et al. Literature research: aims and design of systematic reviews. Aust J Physiother. 2005;51(2):125-8.
27. Debray TPA, Damen JAAG, Snell KIE, et al. A guide to systematic review and meta-analysis of prediction model performance. BMJ (Online). 2017;356.
28. Dijkers MP, Bushnik T, Heinemann AW, et al. Systematic reviews for informing rehabilitation practice: an introduction. Arch Phys Med Rehabil. 2012;93(5):912-8.
29. Fares M, Alahdab F, Alsaied T. Systematic Reviews and Meta-analyses for Cardiology Fellows. Congenit Heart Dis. 2016;11(4):369-71.
30. Gomersall JS, Jadotte YT, Xue Y, et al. Conducting systematic reviews of economic evaluations. Int J Evid Based Healthc. 2015;13(3):170-8.
31. Goodacre S. Critical appraisal for emergency medicine: 6 systematic reviews. Emerg Med J. 2009;26(2):114-6.
32. Guise JM, Chang C, Viswanathan M, et al. Agency for healthcare research and quality evidence-based practice center methods for systematically reviewing complex multicomponent health care interventions. Journal of Clinical Epidemiology. 2014;67(11):1181-91.
33. Guise JM, Chang C, Butler ME, et al. AHRQ series on complex intervention systematic reviews-paper 1: an introduction to a series of articles that provide guidance and tools for reviews of complex interventions. J Clin Epidemiol. 2017;90:6-10.
34. Kelly MP, Noyes J, Kane RL, et al. AHRQ series on complex intervention systematic reviews-paper 2: defining complexity, formulating scope, and questions. J Clin Epidemiol. 2017;90:11-8.
35. Butler M, Epstein RA, Totten A, et al. AHRQ series on complex intervention systematic reviews—paper 3: adapting frameworks to develop protocols. Journal of Clinical Epidemiology. 2017;90:19-27.
36. Viswanathan M, McPheeters ML, Murad MH, et al. AHRQ series on complex intervention systematic reviews—paper 4: selecting analytic approaches. Journal of Clinical Epidemiology. 2017;90:28-36.
37. Pigott T, Noyes J, Umscheid CA, Myers E, Morton SC, Fu R, et al. AHRQ series on complex intervention systematic reviews-paper 5: advanced analytic methods. J Clin Epidemiol. 2017;90:37-42.
38. Guise JM, Butler ME, Chang C, Viswanathan M, Pigott T, Tugwell P. AHRQ series on complex intervention systematic reviews—paper 6: PRISMA-CI extension statement and checklist. Journal of Clinical Epidemiology. 2017;90:43-50.
39. Guise JM, Butler M, Chang C, Viswanathan M, Pigott T, Tugwell P. AHRQ series on complex intervention systematic reviews-paper 7: PRISMA-CI elaboration and explanation. J Clin Epidemiol. 2017;90:51-8.
40. Haase SC. Systematic reviews and meta-analysis. Plast Reconstr Surg. 2011;127(2):955-66.
41. Harris JD, Quatman CE, Manring MM, Siston RA, Flanigan DC. How to write a systematic review. Am J Sports Med. 2014;42(11):2761-8.
42. Henderson LK, Craig JC, Willis NS, Tovey D, Webster AC. How to write a Cochrane systematic review. Nephrology (Carlton). 2010;15(6):617-24.
43. Hoffmann S, de Vries RBM, Stephens ML, Beck NB, Dirven HAAM, Fowle JR, et al. A primer on systematic reviews in toxicology. Archives of Toxicology. 2017;91(7):2551-75.
44. Hopp L, Rittenmeyer L. Review and Synthesize Completed Research Through Systematic Review. West J Nurs Res. 2015;37(10):1359-72.
45. Aromataris E, Munn Z (Editors). Joanna Briggs Institute Reviewer's Manual. The Joanna Briggs Institute, 2017. Available at: https://reviewersmanual.joannabriggs.org/ (last accessed 04 Oct 2018).
46. Jones T, Evans D. Conducting a systematic review. Aust Crit Care. 2000;13(2):66-71.
47. Kalra R, Arora P, Morgan C, et al. Conducting and interpreting high-quality systematic reviews and meta-analyses. Journal of Nuclear Cardiology. 2017;24(2):471-81.
48. Kelley BP, Chung KC. Developing, Conducting, and Publishing Appropriate Systematic Review and Meta-Analysis Articles. Plast Reconstr Surg. 2018;141(2):516-25.
49. Khan KS, Kunz R, Kleijnen J, et al. Five steps to conducting a systematic review. J R Soc Med. 2003;96(3):118-21.
50. Khan KS. Systematic reviews of diagnostic tests: a guide to methods and application. Best Pract Res Clin Obstet Gynaecol. 2005;19(1):37-46.
51. Koretz RL, Lipman TO. Understanding systematic reviews and meta-analyses. Journal of Parenteral and Enteral Nutrition. 2017;41(3):316-23.
52. Kranke P. Evidence-based practice: how to perform and use systematic reviews for clinical decision-making. Eur J Anaesthesiol. 2010;27(9):763-72.
53. Leeflang MM. Systematic reviews and meta-analyses of diagnostic test accuracy. Clin Microbiol Infect. 2014;20(2):105-13.
54. Lipp A. A guide to developing a systematic review. Aorn j. 2003;78(1):90-4, 7-107.
55. Liu Z, Yao Z, Li C, Liu X, Chen H, Gao C. A step-by-step guide to the systematic review and meta-analysis of diagnostic and prognostic test accuracy evaluations. Br J Cancer. 2013;108(11):2299-303.
56. Manchikanti L, Benyamin RM, Helm S, et al. Evidence-based medicine, systematic reviews, and guidelines in interventional pain management: part 3: systematic reviews and meta-analyses of randomized trials. Pain Physician. 2009;12(1):35-72.
57. Manchikanti L, Datta S, Smith HS, Hirsch JA. Evidence-based medicine, systematic reviews, and guidelines in interventional pain management: part 6. Systematic reviews and meta-analyses of observational studies. Pain Physician. 2009;12(5):819-50.
58. Manchikanti L, Derby R, Wolfer L, Singh V, Datta S, Hirsch JA. Evidence-based medicine, systematic reviews, and guidelines in interventional pain management: Part 7: systematic reviews and meta-analyses of diagnostic accuracy studies. Pain Physician. 2009;12(6):929-63.
59. Marchevsky AM, Wick MR. Evidence-based pathology: systematic literature reviews as the basis for guidelines and best practices. Arch Pathol Lab Med. 2015;139(3):394-9.
60. Marshall G, Sykes AE. Systematic reviews: A guide for radiographers and other health care professionals. Radiography. 2011;17(2):158-64.
61. Matchar DB. Chapter 1: Introduction to the Methods Guide for Medical Test Reviews. J Gen Intern Med. 2012;27 Suppl 1:S4-10.
62. Samson D, Schoelles KM. Chapter 2: medical tests guidance (2) developing the topic and structuring systematic reviews of medical tests: utility of PICOTS, analytic frameworks, decision trees, and other frameworks. J Gen Intern Med. 2012;27 Suppl 1:S11-9.
63. Segal JB. Chapter 3: choosing the important outcomes for a systematic review of a medical test. J Gen Intern Med. 2012;27 Suppl 1:S20-7.
64. Relevo R. Chapter 4: Effective search strategies for systematic reviews of medical tests. Journal of General Internal Medicine. 2012;27(SUPPL.1):S28-S32.
65. Santaguida PL, Riley CM, Matchar DB. Chapter 5: assessing risk of bias as a domain of quality in medical test studies. J Gen Intern Med. 2012;27 Suppl 1:S33-8.
66. Hartmann KE, Matchar DB, Chang S. Chapter 6: assessing applicability of medical test studies in systematic reviews. J Gen Intern Med. 2012;27 Suppl 1:S39-46.
67. Singh S, Chang SM, Matchar DB, et al. Chapter 7: grading a body of evidence on diagnostic tests. J Gen Intern Med. 2012;27 Suppl 1:S47-55.
68. Trikalinos TA, Balion CM, Coleman CI, et al. Chapter 8: meta-analysis of test performance when there is a "gold standard". J Gen Intern Med. 2012;27 Suppl 1:S56-66.
69. Trikalinos TA, Balion CM. Chapter 9: options for summarizing medical test performance in the absence of a "gold standard". J Gen Intern Med. 2012;27 Suppl 1:S67-75.
70. Trikalinos TA, Kulasingam S, Lawrence WF. Chapter 10: Deciding Whether to Complement a Systematic Review of Medical Tests with Decision Modeling. J Gen Intern Med. 2012;27(Suppl 1):76-82.
71. Jonas DE, Wilt TJ, Taylor BC, Wilkins TM, Matchar DB. Chapter 11: Challenges in and principles for conducting systematic reviews of genetic tests used as predictive indicators. Journal of General Internal Medicine. 2012;27(SUPPL.1):S83-S93.
72. Rector TS, Taylor BC, Wilt TJ. Chapter 12: systematic review of prognostic tests. J Gen Intern Med. 2012;27 Suppl 1:S94-101.
73. Menzies D. Systematic reviews and meta-analyses. International Journal of Tuberculosis and Lung Disease. 2011;15(5):582-93.
74. Knoll T, Omar MI, Maclennan S, Hernández V, Canfield S, Yuan Y, et al. Key Steps in Conducting Systematic Reviews for Underpinning Clinical Practice Guidelines: Methodology of the European Association of Urology. European Urology. 2018;73(2):290-300.
75. Agency for Healthcare Research and Quality. Methods Guide for Effectiveness and Comparative Effectiveness Reviews. AHRQ Publication No. 10(14)-EHC063-EF. Rockville. 2014. Available at: https://effectivehealthcare.ahrq.gov/sites/default/files/pdf/cer-methods-guide_overview.pdf (last accessed 02 Oct 2018).
76. Milner KA. Systematic reviews. Oncol Nurs Forum. 2015;42(1):89-93.
77. Moola S, Munn Z, Sears K, et al. Conducting systematic reviews of association (etiology): The Joanna Briggs Institute's approach. Int J Evid Based Healthc. 2015;13(3):163-9.
78. Munn Z, Moola S, Lisy K, et al. Methodological guidance for systematic reviews of observational epidemiological studies reporting prevalence and cumulative incidence data. Int J Evid Based Healthc. 2015;13(3):147-53.
79. Neely JG, Magit AE, Rich JT, et al. A practical guide to understanding systematic reviews and meta-analyses. Otolaryngol Head Neck Surg. 2010;142(1):6-14.
80. Nguyen NH, Singh S. A Primer on Systematic Reviews and Meta-Analyses. Seminars in Liver Disease. 2018;38(2):103-11.
81. Nicholson PJ. How to undertake a systematic review in an occupational setting. Occup Environ Med. 2007;64(5):353-8, 03.
82. Noordzij M, Zoccali C, Dekker FW, et al. Adding up the evidence: Systematic reviews and meta-analyses. Nephron - Clinical Practice. 2011;119(4):C310-C6.
83. Pollock A, Berge E. How to do a systematic review. International Journal of Stroke. 2018;13(2):138-56.
84. Moher D, Liberati A, Tetzlaff J, et al. Preferred reporting items for systematic reviews and meta-analyses: the PRISMA statement. Int J Surg. 2010;8(5):336-41.
85. Moher D, Liberati A, Tetzlaff J, et al. Reprint--preferred reporting items for systematic reviews and meta-analyses: the PRISMA statement. Phys Ther. 2009;89(9):873-80.
86. Moher D, Liberati A, Tetzlaff J, et al. Preferred reporting items for systematic reviews and meta-analyses: the PRISMA statement. J Clin Epidemiol. 2009;62(10):1006-12.
87. Liberati A, Altman DG, Tetzlaff J, et al. The PRISMA statement for reporting systematic reviews and meta-analyses of studies that evaluate health care interventions: explanation and elaboration. J Clin Epidemiol. 2009;62(10):e1-34.
88. Liberati A, Altman DG, Tetzlaff J, et al. The PRISMA statement for reporting systematic reviews and meta-analyses of studies that evaluate healthcare interventions: explanation and elaboration. Bmj. 2009;339:b2700.
89. Moher D, Liberati A, Tetzlaff J, et al. Preferred reporting items for systematic reviews and meta-analyses: the PRISMA statement. Bmj. 2009;339:b2535.
90. Liberati A, Altman DG, Tetzlaff J, et al. The PRISMA statement for reporting systematic reviews and meta-analyses of studies that evaluate health care interventions: explanation and elaboration. Ann Intern Med. 2009;151(4):W65-94.
91. Moher D, Liberati A, Tetzlaff J, et al. Preferred reporting items for systematic reviews and meta-analyses: the PRISMA statement. Ann Intern Med. 2009;151(4):264-9, w64.
92. Moher D, Liberati A, Tetzlaff J, et al. Preferred reporting items for systematic reviews and meta-analyses: the PRISMA statement. PLoS Med. 2009;6(7):e1000097.
93. Liberati A, Altman DG, Tetzlaff J, et al. The PRISMA statement for reporting systematic reviews and meta-analyses of studies that evaluate health care interventions: explanation and elaboration. PLoS Med. 2009;6(7):e1000100.
94. Zorzela L, Loke YK, Ioannidis JP, et al. PRISMA harms checklist: improving harms reporting in systematic reviews. Bmj. 2016;352:i157.
95. McInnes MDF, Moher D, Thombs BD, et al. Preferred Reporting Items for a Systematic Review and Meta-analysis of Diagnostic Test Accuracy Studies: The PRISMA-DTA Statement. Jama. 2018;319(4):388-96.
96. Welch V, Petticrew M, Petkovic J, et al. Extending the PRISMA statement to equity-focused systematic reviews (PRISMA-E 2012): explanation and elaboration. J Clin Epidemiol. 2016;70:68-89.
97. Welch V, Petticrew M, Tugwell P, et al. PRISMA-Equity 2012 Extension: Reporting Guidelines for Systematic Reviews with a Focus on Health Equity. PLoS Medicine. 2012;9(10):e1001333. doi:10.1371/journal.pmed.1001333.
98. Welch V, Petticrew M, Petkovic J, et al. Extending the PRISMA statement to equity-focused systematic reviews (PRISMA-E 2012): explanation and elaboration. Int J Equity Health. 2015;14:92.
99. Stewart LA, Clarke M, Rovers M, et al. Preferred reporting items for a systematic review and meta-analysis of individual participant data: The PRISMA-IPD statement. JAMA - Journal of the American Medical Association. 2015;313(16):1657-65.
100. Hutton B, Catalá-López F, Moher D. The PRISMA statement extension for systematic reviews incorporating network meta-analysis: PRISMA-NMA. Medicina Clinica. 2016;147(6):262-6.
101. Hutton B, Salanti G, Caldwell DM, et al. The PRISMA extension statement for reporting of systematic reviews incorporating network meta-analyses of health care interventions: Checklist and explanations. Annals of Internal Medicine. 2015;162(11):777-84.
102. Moher D, Shamseer L, Clarke M, et al. Preferred reporting items for systematic review and meta-analysis protocols (PRISMA-P) 2015 statement. Syst Rev. 2015;4:1.
103. Shamseer L, Moher D, Clarke M, et al. Preferred reporting items for systematic review and meta-analysis protocols (prisma-p) 2015: Elaboration and explanation. BMJ (Online). 2015;349.
104. Ravindran V, Shankar S. Systematic reviews and meta-analysis demystified. 2015;10(2):89-94.
105. Rew L. The systematic review of literature: synthesizing evidence for practice. J Spec Pediatr Nurs. 2011;16(1):64-9.
106. Riesenberg LA, Justice EM. Conducting a successful systematic review of the literature, part 1. Nursing. 2014;44(4):13-7.
107. Riesenberg LA, Justice EM. Conducting a successful systematic review of the literature, part 2. Nursing. 2014;44(6):23-6.
108. Rudnicka AR, Owen CG. An introduction to systematic reviews and meta-analyses in health care. Ophthalmic Physiol Opt. 2012;32(3):174-83.
109. Sambunjak D, Franić M. Steps in the undertaking of a systematic review in orthopaedic surgery. International Orthopaedics. 2012;36(3):477-84.
110. Sayers A. Tips and tricks in performing a systematic review. Br J Gen Pract. 2007;57(538):425.
111. Sayers A. Tips and tricks in performing a systematic review. Br J Gen Pract. 2007;57(542):759.
112. Sayers A. Tips and tricks in performing a systematic review. Br J Gen Pract. 2007;57(545):999.
113. Sayers A. Tips and tricks in performing a systematic review--chapter 4. Br J Gen Pract. 2008;58(547):136.
114. Schweizer ML, Nair R. A practical guide to systematic literature reviews and meta-analyses in infection prevention: Planning, challenges, and execution. Am J Infect Control. 2017;45(11):1292-4.
115. Rao G, Lopez-Jimenez F, Boyd J, et al. Methodological standards for meta-analyses and qualitative systematic reviews of cardiac prevention and treatment studies a scientific statement from the American Heart Association. Circulation. 2017;136(10):e172-e94.
116. Shenkin SD, Harrison JK, Wilkinson T, et al. Systematic reviews: Guidance relevant for studies of older people. Age and Ageing. 2017;46(5):722-8.
117. Sousa MR, Ribeiro AL. Systematic review and meta-analysis of diagnostic and prognostic studies: a tutorial. Arq Bras Cardiol. 2009;92(3):229-38, 35-45.
118. Standards for the design and conduct of systematic reviews with low-level laser therapy for musculoskeletal pain and disorders. Photomed Laser Surg. 2006;24(6):759-60.
119. Staunton M. Evidence-based radiology: steps 1 and 2--asking answerable questions and searching for evidence. Radiology. 2007;242(1):23-31.
120. Halligan S, Altman DG. Evidence-based practice in radiology: steps 3 and 4--appraise and apply systematic reviews and meta-analyses. Radiology. 2007;243(1):13-27.
121. Henry BM, Tomaszewski KA, Walocha JA. Methods of Evidence-Based Anatomy: a guide to conducting systematic reviews and meta-analysis of anatomical studies. Ann Anat. 2016;205:16-21.
122. Thrift AG. Systematic review of observational studies. Neuroepidemiology. 2010;34(4):262-3.
123. Uman LS. Systematic reviews and meta-analyses. Journal of the Canadian Academy of Child and Adolescent Psychiatry. 2011;20(1):57-9.
124. Umscheid CA. A primer on performing systematic reviews and meta-analyses. Clinical Infectious Diseases. 2013;57(5):725-34.
125. van Mastrigt GA, Hiligsmann M, Arts JJ, et al. How to prepare a systematic review of economic evaluations for informing evidence-based healthcare decisions: a five-step approach (part 1/3). Expert Rev Pharmacoecon Outcomes Res. 2016;16(6):689-704.
126. Thielen FW, Van Mastrigt G, Burgers LT, et al. How to prepare a systematic review of economic evaluations for clinical practice guidelines: database selection and search strategy development (part 2/3). Expert Rev Pharmacoecon Outcomes Res. 2016;16(6):705-21.
127. Wijnen B, Van Mastrigt G, Redekop WK, et al. How to prepare a systematic review of economic evaluations for informing evidence-based healthcare decisions: data extraction, risk of bias, and transferability (part 3/3). Expert Rev Pharmacoecon Outcomes Res. 2016;16(6):723-32.
128. Wanden-Berghe C, Sanz-Valero J. Systematic reviews in nutrition: standardized methodology. Br J Nutr. 2012;107 Suppl 2:S3-7.
129. White A, Schmidt K. Systematic literature reviews. Complement Ther Med. 2005;13(1):54-60.
130. Wieseler B, McGauran N. Reporting a systematic review. Chest. 2010;137(5):1240-6.
131. Yanagawa B, Tam DY, Mazine A, et al. Systematic review and meta-analysis in cardiac surgery: A primer. Current Opinion in Cardiology. 2018;33(2):184-9.

**Excluded articles with reasons for exclusion**

Abstract:

Elias FTS, Koury CDN. Publication of methodological guidelines: The development of systematic reviews (SR) and meta-analyses of randomized clinical trials by the department of science and technology of the Brazilian ministry of health (DECIT/MOH). Value in Health. 2014;17(3):A206-A7.

Sun Z. Evidence-based medicine: Tips to conduct systematic reviews and meta-analyses of observational studies. Journal of Medical Imaging and Radiation Oncology. 2013;57:167-8.

Thompson J, Hawkins N. Juggling jurisdictions: Methods for conducting modular systematic reviews? Value in Health. 2013;16(7):A614.

Commentaries:

Anderson NK, Jayaratne YS. Methodological challenges when performing a systematic review. Eur J Orthod. 2015;37(3):248-50.

Beller E, Clark J, Tsafnat G, et al. Making progress with the automation of systematic reviews: Principles of the International Collaboration for the Automation of Systematic Reviews (ICASR). Systematic Reviews. 2018;7(1).

Biondi-Zoccai GGL, Abbate A, Sheiban I. Systematic reviews and meta-analyses "for dummies". EuroIntervention. 2009;5(3):289-91.

Cook DA. Tips for a great review article: crossing methodological boundaries. Med Educ. 2016;50(4):384-7.

Cornell JE. The PRISMA extension for network Meta-analysis: Bringing clarity and guidance to the reporting of systematic reviews incorporating network meta-analyses. Annals of Internal Medicine. 2015;162(11):797-8.

Falck-Ytter Y, Schunemann H, Guyatt G. AHRQ series commentary 1: rating the evidence in comparative effectiveness reviews. J Clin Epidemiol. 2010;63(5):474-5.

Heesen M, Klimek M, Imberger G, et al. On differences between systematic reviews. British Journal of Anaesthesia. 2018;120(5):1133-4.

Hutton B, Moher D, Cameron C. The PRISMA extension statement. Annals of Internal Medicine. 2015;163(7):566-7.

McLeroy KR, Northridge ME, Balcazar H, et al. Reporting guidelines and the American Journal of Public Health's adoption of Preferred Reporting Items for Systematic reviews and Meta-Analyses. Am J Public Health. 2012;102(5):780-4.

Pieper D, Allers K. Differences between protocols for randomized controlled trials and systematic reviews. Journal of Clinical Epidemiology. 2018;98:144-5.

Editorial:

Atallah AN, Puchnick A, Wu D, et al. Remarks about systematic reviews of diagnostic tests. Sao Paulo Med J. 2012;130(5):279-81.

Baker JD. The Purpose, Process, and Methods of Writing a Literature Review. Aorn j. 2016;103(3):265-9.

Bannigan K. Systematic review: The first step in developing a complex intervention. JBI Database of Systematic Reviews and Implementation Reports. 2018;16(5):1079-80.

Blegen MA. PRISMA. Nurs Res. 59. United States2010. p. 233.

Caldwell DM. An overview of conducting systematic reviews with network meta-analysis. Syst Rev. 3. England2014. p. 109.

Chung KC. JHS guidelines on systematic review and meta-analysis submissions. J Hand Surg Am. 37. United States2012. p. 1121-4.

Citrome L. Systematic reviews: much ado about a lot. Int J Clin Pract. 63. England2009. p. 832-3.

Harvey L, Wyndaele JJ. Reporting and interpreting results of systematic reviews. Spinal Cord. 47. England2009. p. 777.

Koelemay MJ, Vermeulen H. Quick Guide to Systematic Reviews and Meta-analysis. Eur J Vasc Endovasc Surg. 2016;51(2):309.

Koop PM, Burgess-Pinto E. Conducting a systematic literature review: a brief guide. Can Oncol Nurs J. 2002;12(4):196-7.

Lowe NK. Systematic literature reviews. J Obstet Gynecol Neonatal Nurs. 38. United States2009. p. 375-6.

Moher D, Stewart L, Shekelle P. Implementing PRISMA-P: recommendations for prospective authors. Syst Rev. 5. England2016. p. 15.

Riemann D. Focus on systematic reviews and insomnia. Journal of Sleep Research. 2018;27(3).

Sharifabadi AD, McInnes MDF, Bossuyt PMM. PRISMA-DTA: An extension of PRISMA for reporting of diagnostic test accuracy systematic reviews. Clinical Chemistry. 2018;64(6):985-6.

Smetana GW, Umscheid CA, Chang S, et al. Methods guide for authors of systematic reviews of medical tests: a collaboration between the Agency for Healthcare Research and Quality (AHRQ) and the Journal of General Internal Medicine. J Gen Intern Med. 2012;27 Suppl 1:S1-3.

Swartz MK. The PRISMA statement: a guideline for systematic reviews and meta-analyses. J Pediatr Health Care. 25. United States2011. p. 1-2.

Tugwell P, Knottnerus JA, McGowan J, et al. Systematic Review Qualitative Methods Series reflect the increasing maturity in qualitative methods. Journal of Clinical Epidemiology. 2018;97:vii-viii.

Turpin DL. CONSORT and QUOROM guidelines for reporting randomized clinical trials and systematic reviews. Am J Orthod Dentofacial Orthop. 128. United States2005. p. 681-5; discussion 6.

Verhagen AP. The art of systematic reviews. Musculoskeletal Science and Practice. 2017;31:iv-vi.

Wikoff DS, Miller GW. Systematic reviews in toxicology. Toxicological Sciences. 2018;163(2):335-7.

Wood MJ. Systematic literature reviews. Clin Nurs Res. 2003;12(1):3-7.

Wright JG, Swiontkowski MF, Tolo VT. Meta-Analyses and Systematic Reviews: New Guidelines for JBJS. J Bone Joint Surg Am. 94. United States2012. p. 1537.

Letter:

Lerner F, Hamblen JL. Methodology and reporting of systematic reviews and meta-analyses. Br J Psychiatry. 202. England2013. p. 75-6.

Nibali L. Suggested guidelines for systematic reviews of periodontal genetic association studies. J Clin Periodontol. 2013;40(8):753-6.

Parry RH, Land V. Systematically reviewing and synthesizing evidence from conversation analytic and related discursive research to inform healthcare communication practice and policy: an illustrated guide. BMC Med Res Methodol. 13. England2013. p. 69.

Literature review (not recommendations):

Adams CE, Polzmacher S, Wolff A. Systematic reviews: Work that needs to be done and not to be done. Journal of Evidence-Based Medicine. 2013;6(4):232-5.

Anderson WG, McNamara MC, Arnold RM. Systematic reviews and meta-analyses. J Palliat Med. 2009;12(10):937-46.

Berlin JA, Cepeda MS. Some methodological points to consider when performing systematic reviews in comparative effectiveness research. Clinical Trials. 2012;9(1):27-34.

Brugha TS, Matthews R, Morgan Z, et al. Methodology and reporting of systematic reviews and meta-analyses of observational studies in psychiatric epidemiology: Systematic review. British Journal of Psychiatry. 2012;200(6):446-53.

Chambers D, Wilson P. A framework for production of systematic review based briefings to support evidence-informed decision-making. Syst Rev. 2012;1:32.

Furlan JC, Singh J, Hsieh J, et al. Methodology of systematic reviews and recommendations. Journal of Neurotrauma. 2011;28(8):1335-9.

Green S. Systematic reviews and meta-analysis. Singapore Med J. 2005;46(6):270-3; quiz 4.

Lodge M. Conducting a systematic review: Finding the evidence. Journal of Evidence-Based Medicine. 2011;4(2):135-9.

Mandrekar JN, Mandrekar SJ. Systematic reviews and meta-analysis of published studies: an overview and best practices. J Thorac Oncol. 2011;6(8):1301-3.

McGrath TA, Alabousi M, Skidmore B, et al. Recommendations for reporting of systematic reviews and meta-analyses of diagnostic test accuracy: a systematic review. Syst Rev. 2017;6(1):194.

Mittal N, Goyal M, Mittal PK. Understanding and Appraising Systematic Reviews and Meta-Analysis. J Clin Pediatr Dent. 2017;41(5):317-26.

Paone S, Di Tanna GL, Corio M, et al. Assessing the methods for systematic reviews of economic evaluations. Value in Health. 2011;14(7):A423.

Pearson A, White H, Bath-Hextall F, et al. A mixed-methods approach to systematic reviews. Int J Evid Based Healthc. 2015;13(3):121-31.

Petticrew M, Rehfuess E, Noyes J, et al. Synthesizing evidence on complex interventions: how meta-analytical, qualitative, and mixed-method approaches can contribute. J Clin Epidemiol. 2013;66(11):1230-43.

Pope JE, Hazlewood GS. Randomized Trials, Meta-Analyses, and Systematic Reviews: Using Examples from Rheumatology. Rheumatic Disease Clinics of North America.

Price B. Guidance on conducting a literature search and reviewing mixed literature. Nurs Stand. 2009;23(24):43-9; quiz 50, 2.

Sipe TA, Chin HB, Elder R, et al. Methods for conducting community guide systematic reviews of evidence on effectiveness and economic efficiency of group-based behavioral interventions to prevent adolescent pregnancy, human immunodeficiency virus, and other sexually transmitted infections: Comprehensive risk reduction and abstinence education. American Journal of Preventive Medicine. 2012;42(3):295-303.

Smith CJ. Systematic reviews and meta-analyses. Phlebology. 2011;26(6):271-3.

Squires JE, Valentine JC, Grimshaw JM. Systematic reviews of complex interventions: Framing the review question. Journal of Clinical Epidemiology. 2013;66(11):1215-22.

Tonelli M, Hackam D, Garg AX. Primer on systematic review and meta-analysis. Methods Mol Biol. 2009;473:217-33.

Vrabel M. Preferred Reporting Items for Systematic Reviews and Meta-Analyses. Oncol Nurs Forum. 2015;42(5):552-4.

Without access:

Arbesman M, Lieberman D, Berlanstein DR. Method for the systematic reviews on occupational therapy and driving and community mobility for older adults. Am J Occup Ther. 2014;68(6):657-61.

Arbesman M, Lieberman D, Berlanstein DR. Method for the systematic reviews on occupational therapy and neurodegenerative diseases. Am J Occup Ther. 2014;68(1):15-9.

Arbesman M, Lieberman D, Berlanstein DR. Method for the systematic reviews on occupational therapy and early intervention and early childhood services. Am J Occup Ther. 2013;67(4):389-94.

Arbesman M, Lieberman D. Methodology for the systematic reviews on occupation- and activity-based intervention related to productive aging. Am J Occup Ther. 2012;66(3):271-6.

Bennett S, Hoffmann T, McCluskey A, et al. Systematic reviews informing occupational therapy. Am J Occup Ther. 2013;67(3):345-54.

Connelly LM. Reviewing the literature. Medsurg Nurs. 2010;19(4):245-6.

Connelly LM. Systematic reviews. Medsurg Nurs. 2009;18(3):181-2.

MacDonald-Jankowski DS, Dozier MF. Systematic review in diagnostic radiology. Dentomaxillofac Radiol. 2001;30(2):78-83.

Pai M, McCulloch M, Gorman JD, et al. Systematic reviews and meta-analyses: an illustrated, step-by-step guide. Natl Med J India. 2004;17(2):86-95.

Overviews:

Botelho RV, Oliveira MF. Guidelines for Integration of Systematic Reviews using Primary Studies. Brazilian Neurosurgery. 2016;35(3):179-84.

Bougioukas KI, Liakos A, Tsapas A, et al. Preferred reporting items for overviews of systematic reviews including harms checklist: a pilot tool to be used for balanced reporting of benefits and harms. Journal of Clinical Epidemiology. 2018;93:9-24.

Lunny C, Brennan SE, McDonald S, et al. Evidence map of studies evaluating methods for conducting, interpreting and reporting overviews of systematic reviews of interventions: Rationale and design. Systematic Reviews. 2016;5(1).

Robinson KA, Chou R, Berkman ND, et al. Twelve recommendations for integrating existing systematic reviews into new reviews: EPC guidance. Journal of Clinical Epidemiology. 2016;70:38-44.

Smith V, Devane D, Begley CM, et al. Methodology in conducting a systematic review of systematic reviews of healthcare interventions. BMC Med Res Methodol. 2011;11(1):15.

Systematic reviews of not healthcare interventions:

Akl EA, Meerpohl JJ, Elliott J, et al. Living systematic reviews: 4. Living guideline recommendations. Journal of Clinical Epidemiology. 2017;91:47-53.

Anderson LM, Fielding JE, Fullilove MT, et al. Methods for conducting systematic reviews of the evidence of effectiveness and economic efficiency of interventions to promote healthy social environments. Am J Prev Med. 2003;24(3 Suppl):25-31.

Elliott JH, Synnot A, Turner T, et al. Living systematic review: 1. Introduction—the why, what, when, and how. Journal of Clinical Epidemiology. 2017;91:23-30.

Whitlock EP, Lopez SA, Chang S, et al. AHRQ series paper 3: identifying, selecting, and refining topics for comparative effectiveness systematic reviews: AHRQ and the effective health-care program. J Clin Epidemiol. 2010;63(5):491-501.

Veterinary:

Sargeant JM, O'Connor AM. Conducting systematic reviews of intervention questions II: Relevance screening, data extraction, assessing risk of bias, presenting the results and interpreting the findings. Zoonoses Public Health. 2014;61 Suppl 1:39-51.

Qualitative evidence:

Lockwood C, Munn Z, Porritt K. Qualitative research synthesis: methodological guidance for systematic reviewers utilizing meta-aggregation. Int J Evid Based Healthc. 2015;13(3):179-87.
